# Dramatic response of synovitis, acne, pustulosis, hyperostosis, and osteitis syndrome to tofacitinib monotherapy: a case report

**DOI:** 10.1186/s13256-024-04366-w

**Published:** 2024-02-16

**Authors:** Stéphanie Dierckx, Jean-François Nisolle, Yves Boutsen

**Affiliations:** 1 Department of Rheumatology, CHU UCL Namur, Mont-Godinne, 1 Avenue Gaston Therasse, 5530 Yvoir, Belgium; 2 Department of Radiology, CHU UCL Namur, Mont-Godinne, 1 Avenue Gaston Therasse, 5530 Yvoir, Belgium

**Keywords:** SAPHO syndrome, Case report, JAK-inhibitors, Tofacitinib

## Abstract

**Introduction:**

The synovitis, acne, pustulosis, hyperostosis, and osteitis (SAPHO) syndrome is a rare condition. Its treatment remains a challenge for clinicians, and often yields mixed results.

**Case:**

We report the case of a 51-year-old Caucasian woman who presented with SAPHO syndrome with mainly axial involvement. She had been treated with sulfasalazine and anti-inflammatory drugs for many years without any success. A few weeks after starting treatment with tofacitinib, both clinical and biological parameters dramatically improved. Imaging also showed considerable regression of the vertebral and pelvic lesions. However, tofacitinib had to be discontinued due to the occurrence of pulmonary embolism. Consequently, recurrence of bone pain and biologic inflammation was rapidly observed.

**Conclusions:**

Anti-JAKs are an interesting treatment option in the management of SAPHO syndrome that need further clinical trials and assessment for validating response.

## Introduction

The synovitis, acne, pustulosis, hyperostosis, and osteitis (SAPHO) syndrome is a rare condition and may not be recognised early due to the heterogeneity of clinical presentation.

Although many treatments have been tested, few of them have shown promising results so far.

Herein, we report the case of a patient who responded clinically, biologically, and radiologically to anti-Janus kinase (JAK) therapy.

## Case report

A 51-year-old Caucasian woman presented to the outpatient clinic with spinal, pelvic, and sternal inflammatory pain lasting for many years. The symptoms had a strong negative impact on her quality of life. She did not present palmoplantar pustulosis. There was no context of fever.

She had been diagnosed with synovitis, acne, pustulosis, hyperostosis, and osteitis (SAPHO) syndrome at the age of 40 years. She had received sulfasalazine and nonsteroidal anti-inflammatory drugs (NSAIDs) for many years with incomplete pain relief.

She also had a history of cervical and lumbar arthrodesis, hypertension, and thyroidectomy.

**Fig. 1:**
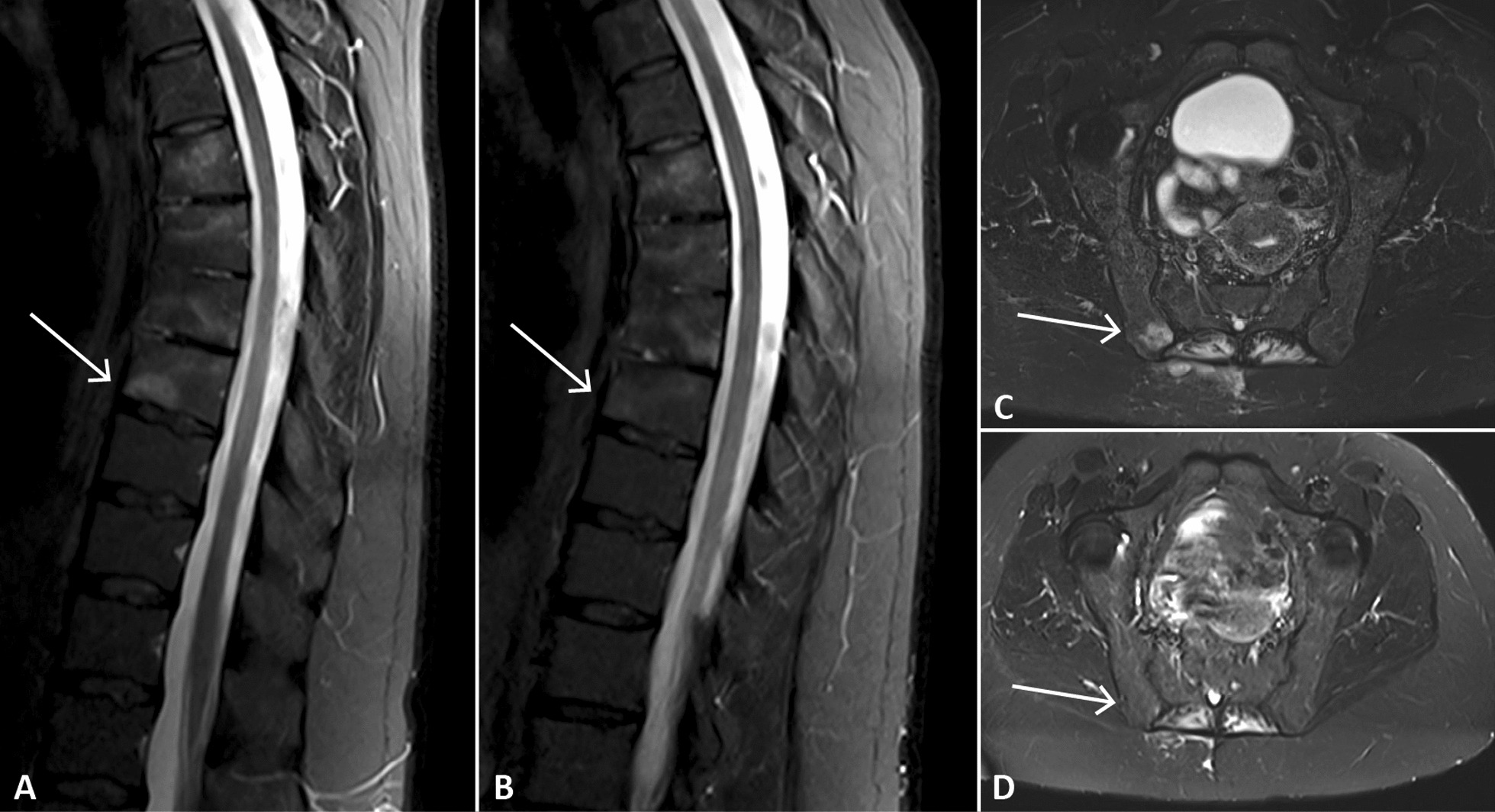
**A**, **B** Magnetic resonance imaging (MRI) of the thoracic spine before and 3 months after the onset of treatment with tofacitinib. **C**, **D** MRI of the iliac bone before and 3 months after the onset of treatment with tofacitinib. Arrows show the bone marrow oedema

**Fig. 2:**
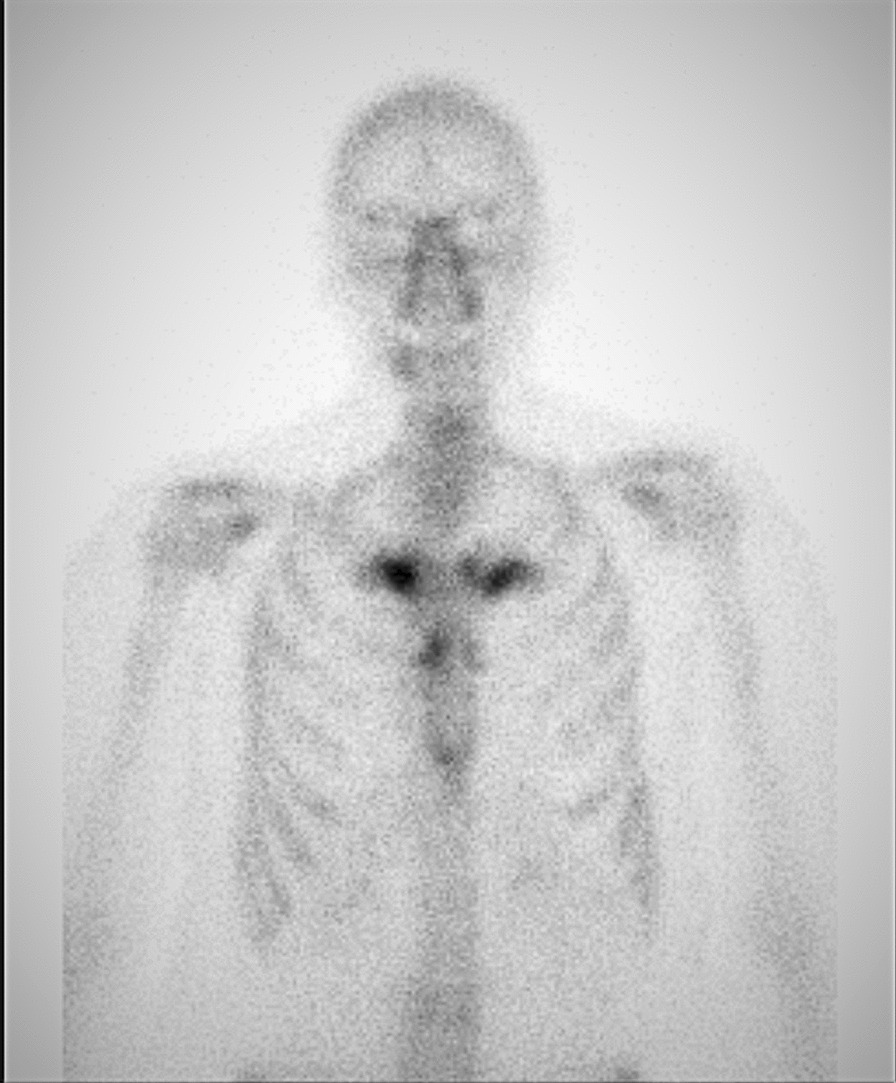
Bone scintigraphy shows increased uptake in the manubrium and sternoclavicular joints

Total spine and pelvis magnetic resonance imaging (MRI) showed a short tau inversion recovery (STIR) hypersignal on several thoracic vertebrae (T6, T7, T8, and T9) and on the sternal part of the right clavicle. Bone marrow oedema was also found on the right iliac bone, though the sacroiliac joints were preserved (Fig. 1). Bone scintigraphy showed anterior chest wall involvement (Fig. 2) with right sternoclavicular hyperostosis on computed tomography.

The C-reactive protein (CRP) was found to be 89.2 mg/L (reference range, 0–5 mg/L). Several blood tests performed earlier had already shown elevation of inflammation parameters. HLA B27 antigen was negative.

We confirmed the diagnosis of active SAPHO syndrome.

We did not consider administering conventional synthetic disease-modifying antirheumatic drug (csDMARD) as methotrexate, which is more effective on peripheral arthritis. Indeed, methotrexate has not shown comparable activity in axial disease in patients with axial spondyloarthritis.

Moreover, we could not prescribe anti-tumour necrosis factor (TNF) drugs because the criteria for reimbursement of these drugs in Belgium were not met in the absence of sacroiliitis.

Given some encouraging results reported with JAK-inhibitors in the literature [1, 2], we initiated tofacitinib 5 mg twice daily.

The patient reported rapid and significant reduction of pain within weeks of starting the treatment.

**Fig. 3:**
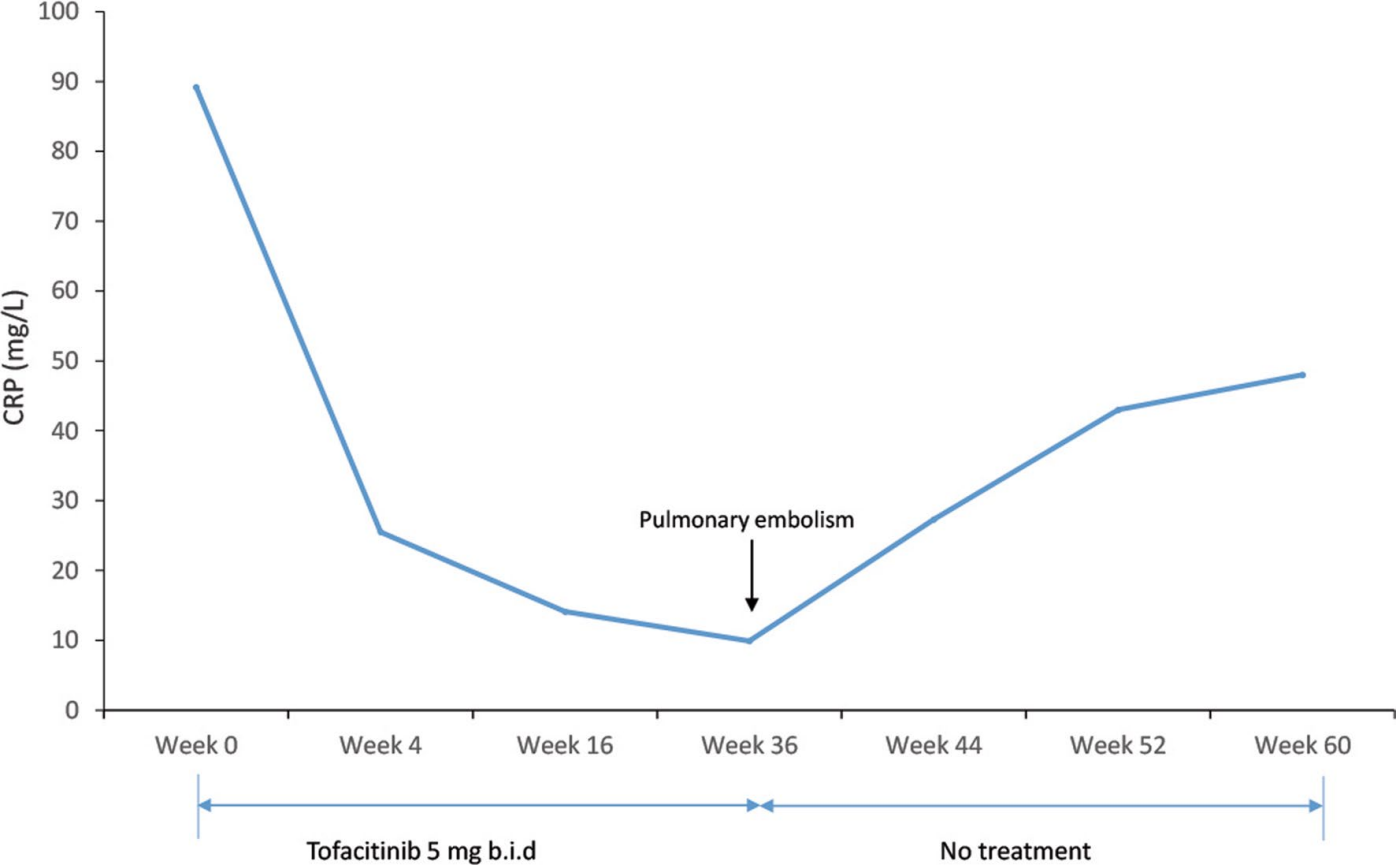
CRP levels evolution with and without tofacitinib treatment. CRP: C-reactive protein; b.i.d: twice per day; SAPHO: synovitis, acne, pustulosis, hyperostosis, and osteitis

Blood tests performed one month after the onset of treatment showed a clear regression of inflammatory parameters, with a CRP at 25.5 mg/L (reference range, 0–5 mg/L), followed by 14 mg/L and 9.9 mg/L after 4 and 10 months of treatment, respectively (Fig. 3).

Total spine and pelvis MRI performed after 3 months of treatment showed regression of the STIR hypersignal on the body of the thoracic vertebrae, as well as the disappearance of the hypersignal of the right iliac bone (Fig. 1).

We could therefore conclude that the rheumatic evolution was favourable from a clinical, biological, and radiological point of view since the introduction of a JAK-inhibitor.

Unfortunately, the treatment had to be discontinued due to pulmonary embolism occurring after 8 months on tofacitinib.

Gradually, spinal, pelvic, and chest (sternal and costal) pain reappeared along with the elevation of inflammatory parameters.

Written informed consent was obtained from the patient for the publication of this report and its accompanying images.

## Discussion

The SAPHO syndrome may have a variable and sometimes incomplete presentation [3]. Skin lesions may have been present for several years before the onset of joint manifestations and forgotten about, or may occur later [4]. Khan *et al.* [5] reported two cases where a 20-year interval was observed between skin and bone involvement, illustrating the heterogeneity of the disease.

As explained by Firinu *et al.* [6], anterior chest wall involvement, mainly that of the sternum and clavicles, is typical of SAPHO syndrome. In terms of spinal involvement, the lesions preferentially affect the dorsal spine [7]. Sacroiliitis can also be observed with variable frequency and is often unilateral with lesions on the iliac side of the joint [8]. In this case, she presented anterior chest wall involvement, dorsal spine lesions and a right iliac bone lesion. There was no sacroiliac joint erosion, which is more specific of spondyloarthritis.

The treatment of SAPHO syndrome remains a challenge. Therapeutic recommendations are based on data collected from small case series, case reports, and expert opinions, but there are no precise guidelines due to rarity of the condition and lack of any randomized trials as a result [3, 4].

NSAIDS and analgesics are recommended as the first line of treatment [6]. In this case, the patient had been using anti-inflammatory drugs for several years without any noticeable benefit. Long-term use of glucocorticoids is not recommended due to the risk of potential complications.

The use of csDMARDs such as methotrexate, sulfasalazine, leflunomide, and azathioprine have shown inconstant results [4, 6]. In our patient, sulfasalazine was ineffective. We did not propose a second csDMARD such as methotrexate because the lesions were essentially axial, without peripheral arthritis.

Anti-TNF drugs are sometimes successful. However, it seems that their efficacy is lower than that noted for other inflammatory rheumatic diseases. It should therefore be reserved for refractory cases [4, 9]. In Belgium, reimbursement of anti-TNFs for SAPHO syndrome requires the presence of sacroiliitis, which was not the case.

Anti-IL23 and anti-IL17 have also been suggested for patients refractory to csDMARDs and other biologic DMARDs. A study reported improvement of skin lesions in 50% of cases; though no benefit was noted for the rheumatic aspects. However, this data was obtained from a small cohort of six patients [10].

Bisphosphonates are frequently included in the list of treatment options; again, with a variable effect [8]. Among them, pamidronate has been studied the most. It is usually proposed as the first line of therapy [11]; however, Gignard *et al.* [12] reported the case of five patients refractory to standard therapy who received bisphosphonates.

Data on the effect of JAK-inhibitors on SAPHO syndrome are currently sparse in the literature. In 2020, a pilot study of 12 patients treated with tofacitinib showed significant improvements [13]. A combination of tofacitinib and methotrexate has been reported in 2 patients [1, 2] with clinical, biological and radiological favourable outcomes. Recently, a case of SAPHO syndrome complicated by ankylosing spondylitis successfully treated with tofacitinib was published [14].

Tofacitinib is an Janus kinase 1/3 inhibitor that demonstrated its efficacy in many rheumatic diseases. As mentioned by Xie *et al.* [15], several studies suggested the involvement of cytokines such as TNF-α, IL-1β, IL-8, IL-17 and IL-18 in the pathogenesis of SAPHO syndrome. They reported the potential effect of tofacitinib and other JAK inhibitors by modulating the cytokine network. Another potential mechanism they highlighted is the suppression of osteoclast-mediated bone resorption by inhibiting the receptor activator for nuclear factor kB ligand (RANKL) pathway.

We reported the case of a patient suffering from SAPHO syndrome who had an excellent response to tofacitinib monotherapy. Both painful symptomatology and CRP increase rapidly reappeared when tofacitinib was discontinued.

## Conclusion

This case is interesting because of the excellent and rapid clinical response to anti-JAK inhibitor monotherapy, as well as the favourable evolution of serum inflammatory parameters and imaging findings. These results are encouraging, considering that few treatments have shown clear efficacy in SAPHO syndrome to date.

## Data Availability

All medical data are available on the hospital server.
